# Three-steps in one-pot: whole-cell biocatalytic synthesis of enantiopure (+)- and (−)-pinoresinol via kinetic resolution

**DOI:** 10.1186/s12934-016-0472-0

**Published:** 2016-05-09

**Authors:** Esther Ricklefs, Marco Girhard, Vlada B. Urlacher

**Affiliations:** Institute of Biochemistry, Heinrich-Heine University, Universitätsstraße 1, 40225 Düsseldorf, Germany

**Keywords:** Laccase, Vanillyl-alcohol oxidase, Pinoresinol reductase, Pinoresinol lariciresinol reductase, Eugenol, Coniferyl alcohol, Pinoresinol, Lignan, Biocatalysis, Kinetic resolution

## Abstract

**Background:**

Pinoresinol is a high-value plant-derived lignan with multiple health supporting effects. Enantiomerically pure pinoresinol can be isolated from natural sources, but with low efficiency. Most chemical and biocatalytic approaches that have been described for the synthesis of pinoresinol furnish the racemic mixture. In this study we devised a three-step biocatalytic cascade for the production of enantiomerically pure pinoresinol from the cheap compound eugenol. Two consecutive oxidations of eugenol through vanillyl-alcohol oxidase and laccase are followed by kinetic resolution of racemic pinoresinol by enantiospecific pinoresinol reductases.

**Results:**

The addition of the enantiospecific pinoresinol reductase from *Arabidopsis thaliana* for kinetic resolution of (±)-pinoresinol to an in vitro cascade involving the vanillyl-alcohol oxidase from *Penicillium simplicissimum* and the bacterial laccase CgL1 from *Corynebacterium glutamicum* resulted in increasing ee values for (+)-pinoresinol; however, an ee value of 34 % was achieved in the best case. The ee value could be increased up to ≥99 % by applying *Escherichia coli*-based whole-cell biocatalysts. The optimized process operated in a one-pot “two-cell” sequential mode and yielded 876 µM (+)-pinoresinol with an ee value of 98 %. Switching the reductase to the enantiospecific pinoresinol lariciresinol reductase from *Forsythia intermedia* enabled the production of 610 µM (−)-pinoresinol with an ee value of 97 %.

**Conclusion:**

A new approach for the synthesis of enantiomerically pure (+)- and (−)-pinoresinol is described that combines three biotransformation steps in one pot. By switching the reductase in the last step, the whole-cell biocatalysts can be directed to produce either (+)- or (−)-pinoresinol. The products of the reductases’ activity, (−)-lariciresinol and (−)-secoisolariciresinol, are valuable precursors that can also be applied for the synthesis of further lignans.

**Electronic supplementary material:**

The online version of this article (doi:10.1186/s12934-016-0472-0) contains supplementary material, which is available to authorized users.

## Background

The phytoestrogen pinoresinol **3** consists of two monolignol units and belongs to the class of lignans. Multiple health supporting effects of pinoresinol **3** have been reported including prevention and/or treatment of cancer [1–3], hyperglycaemia [4], HIV [3], skin-pigmentation [5], microvascular damage [6], and fungal infections [7]. Besides that, pinoresinol **3** is a precursor of the mammalian lignans enterodiol and enterolactone, for which health supporting effects were also reported [8–10]. Additionally, pinoresinol **3** can be used as antifungal agent for the treatment of *Fusarium* head blight causing high mycotoxin levels in wheat [11].

Currently, pinoresinol **3** is mainly isolated from seeds, fruits, and vegetables with low efficiency, but sometimes with high enantiopurity [12–14]. For example, 15 kg perisperm of *Sesamum indicum* are required for isolation of 162 mg enantiopure (+)-pinoresinol **3a**, or 114 g *Daphne odora* for 20.6 mg (−)-pinoresinol **3b** [12, 14].

Additionally, a number of chemical and enzymatic approaches for the synthesis of (±)-pinoresinol **3** have been described. Generally, these synthetic approaches start from simple and abundant low-value compounds (for example methyl acetoacetate), but require multiple steps and intensive work-up [15]. Alternatively, the number of required steps can be reduced by the use of more complex, but rare and expensive starting compounds (for example coniferyl alcohol **2**) [16]. The absence of an inexpensive production process and the large number of potential applications make pinoresinol **3** a high-value compound with growing interest from an economic point of view.

Recently, we have described an in vitro two-step one-pot biocatalytic route for the synthesis of (±)-pinoresinol **3** starting from the inexpensive substrate eugenol **1** [17]. This one-pot cascade combines the vanillyl-alcohol oxidase from *Penicillium simplicissimum* (PsVAO) that converts eugenol **1** into the intermediate coniferyl alcohol **2** and a bacterial laccase for oxidation of coniferyl alcohol **2** leading to (±)-pinoresinol **3**. The best results were achieved with the laccase CgL1 from *Corynebacterium glutamicum*. In the present study we describe the implementation of this cascade into whole-cell biocatalysts and the addition of a third enzymatic step allowing the synthesis of enantiopure (+)-pinoresinol **3a** or (−)-pinoresinol **3b**.

Generally, two strategies are possible for the production of enantiopure pinoresinol **3**: (1) Addition of a dirigent protein, or (2) kinetic resolution of (±)-pinoresinol **3**. Lewis and colleagues demonstrated that dirigent proteins are responsible for enantioselective production of pinoresinol **3** in plants [18, 19]. The mechanism of their action has not been elucidated in detail yet, but it is assumed that dirigent proteins capture the radicals of oxidized coniferyl alcohol **2** and give them a defined orientation for coupling [20]. Therefore, the application of dirigent proteins for selective oxidative phenol coupling seems attractive, but is hampered by the fact that the expression levels achieved in recombinant hosts (e.g. *Pichia pastoris, Solanum peruvianum, Drosophila melanogaster*) are very low [21–23] and that their isolation from natural sources is not feasible [24, 25]. Furthermore, it has been described that the addition of dirigent proteins to in vitro reactions for the synthesis of (+)-pinoresinol **3a** from coniferyl alcohol **2** leads to improved ee values of maximum ~86 % [22, 23, 26].

**Scheme 1:**
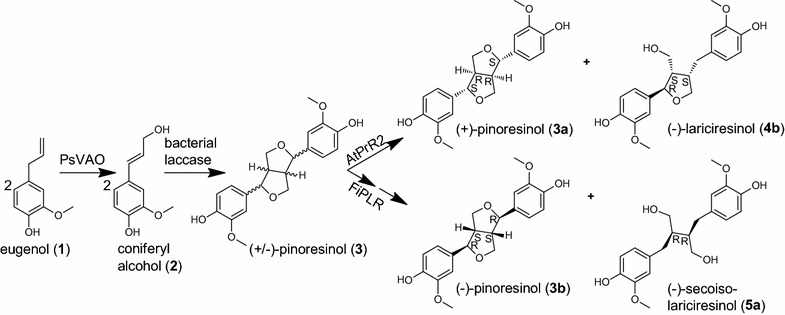
Synthesis of enantiopure pinoresinol **3** by a three-step cascade reaction. The one-pot synthesis combines PsVAO, a bacterial laccase, and an enantiospecific pinoresinol reductase

We decided to use an enantiospecific enzyme for kinetic resolution of (±)-pinoresinol **3**. Pinoresinol reductases (PrR) and pinoresinol lariciresinol reductases (PLR) are NADPH-dependent enzymes capable of reducing pinoresinol **3** to lariciresinol **4** [27, 28]. In a second step PLR can further reduce lariciresinol **4** to secoisolariciresinol **5** [27]. Both, lariciresinol **4** and secoisolariciresinol **5**, are high-value compounds. Almost all PrRs and PLRs characterized so far originate from plants [27–32], except two PrRs that were discovered in sphingomonads [33]. Some PrRs and PLRs were reported to display enantioselectivity: PrR from *Arabidopsis thaliana* (NCBI Reference Sequence: NP_193102.1; AtPrR2) converts preferably (−)-pinoresinol **3b** to (−)-lariciresinol **4b** [28], while PLR from *Forsythia intermedia* (GenBank AAC49608; FiPLR) reduces preferably (+)-pinoresinol **3a** to (+)-lariciresinol **4a** [and further to (−)-secoisolariciresinol **5a**] [27]. In this work we investigated the potential of kinetic resolution for the enrichment of enantiopure (+)-pinoresinol **3a** by AtPrR2 and (−)-pinoresinol **3b** by FiPLR. This step was incorporated into a one-pot three-step synthesis starting from the inexpensive substrate eugenol **1** (Scheme 1).

## Results and discussion

### Expression of reductases

**Fig. 1:**
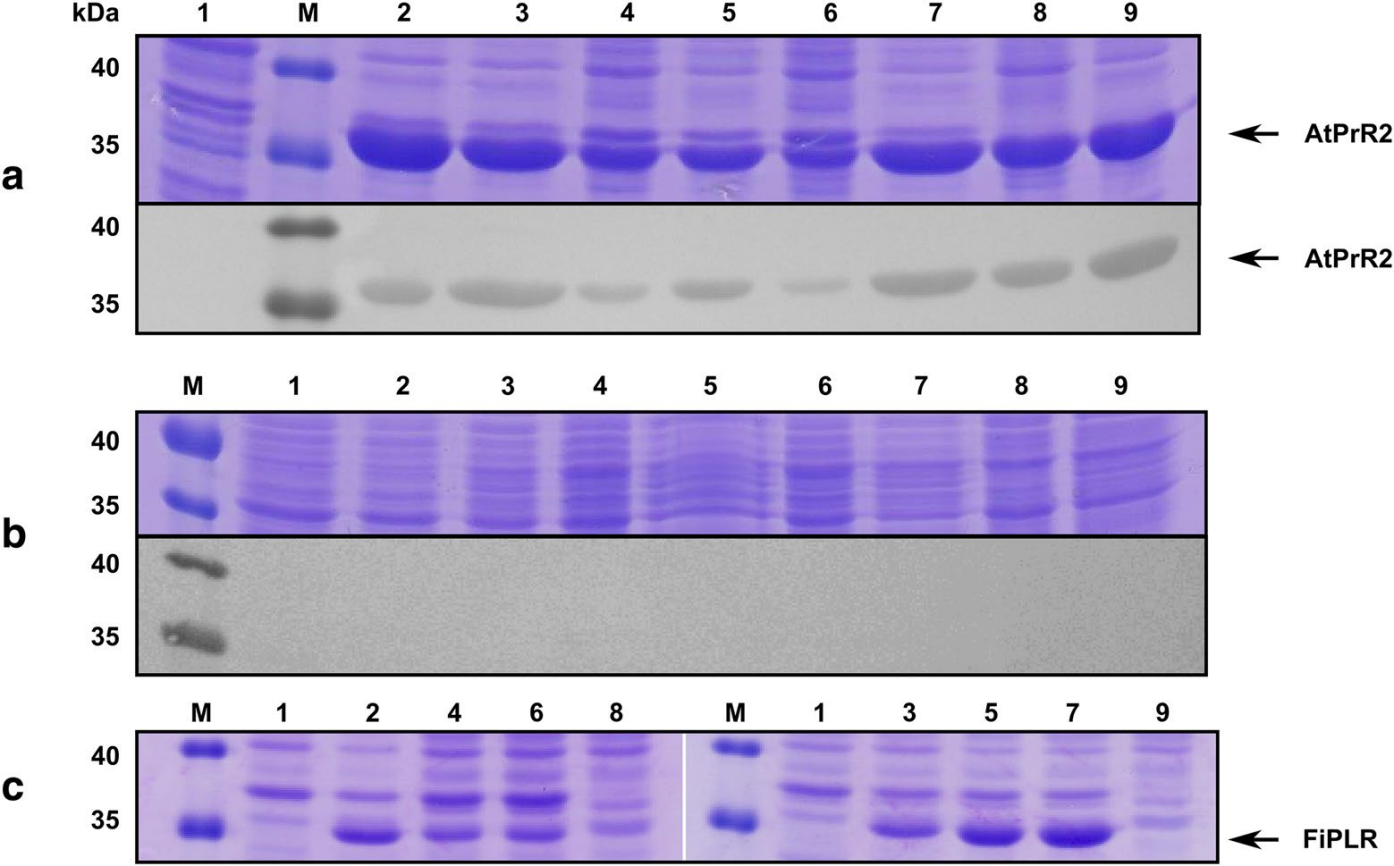
SDS-PAGE and Western-Blot analysis of reductase gene expressions in different *E.* *coli* strains. **a** Expression of *syatprr*2 with C-terminal His_6_-tag; **b** expression of *syfiplr* with C-terminal His_6_-tag; **c** expression of *syfiplr* without His_6_-tag. *1* *E.* *coli* BL21(DE3) cells before induction, *2* *E.* *coli* BL21(DE3) cells 6 h, *3* *E.* *coli* BL21(DE3) cells 24 h, *4* *E.* *coli* C41(DE3) cells 6 h, *5* *E.* *coli* C41(DE3) cells 24 h, *6* *E.* *coli* C43(DE3) cells 6 h, *7* *E.* *coli* C43(DE3) cells 24 h, *8* *E.* *coli* SHuffle T7 Express cells 6 h, *9* *E.* *coli* SHuffle T7 Express cells 24 h, *M* Molecular weight marker

The genes *syatprr*2 and *syfiplr* coding for the reductases AtPrR2 and FiPLR, respectively, were cloned and expressed in recombinant *Escherichia coli*. In order to achieve high expression levels of the reductases, codon optimized genes (see Additional file 1) were used, and the expression in several *E.* *coli* strains was compared (Fig. 1).

SDS-PAGE and Western-Blot revealed that both reductases could be expressed in a soluble form (Fig. 1). Activity assays with pinoresinol **3** (see “Methods” section) demonstrated that the highest conversions were achieved when the soluble protein fractions (cleared cell lysates) after the heterologous expressions of AtPrR2 and FiPLR in *E.* *coli* strains C41(DE3) or C43(DE3) were deployed (see Additional file 2).

### In vitro one-pot three-step cascade reaction for the synthesis of enantiopure pinoresinol

In a first trial to synthesize enantiopure pinoresinol **3**, the vanillyl-alcohol oxidase PsVAO and the bacterial laccase CgL1 used in the previously established one-pot cascade were combined with AtPrR2 from *A.* *thaliana* and tested in vitro under the conditions best suited for the first two bioconversion steps [17]. Unexpectedly, no reduction of (−)-pinoresinol **3b** to (−)-lariciresinol **4b** was observed under these conditions (ee = 0 %; data not shown). Also a sequential reaction set-up (addition of AtPrR2 to the PsVAO-CgL1 cascade after 22 h) resulted only in a minor conversion of (−)-pinoresinol **3b**; the achieved ee value of the remaining (+)-pinoresinol **3a** was 34 %. A prolonged reaction time did not lead to increased ee values. As *tert*-butylmethylester (*t*BME) was added to the previously established PsVAO-CgL1 cascade to enhance the yield of (±)-pinoresinol **3**, we supposed that this organic solvent could negatively affect AtPrR2 activity. Indeed, when we set up the activity assay for conversion of pinoresinol **3** by AtPrR2 in the presence of *t*BME, the pinoresinol **3** conversion was decreased (53 % with *t*BME vs 98 % without *t*BME), which allows the assumption that AtPrR2 is not stable in the presence of this organic solvent. In addition, it was found that the presence of eugenol **1** also negatively affects the conversion of pinoresinol **3** by AtPrR2 (only 60 % conversion). Obviously, the use of the selected isolated enzymes in an in vitro one-pot mode is not suitable to achieve high enantiopurity of pinoresinol **3**.

### Laccase screening for establishment of whole-cell biocatalysts

Based on these results, in the next set of experiments the focus was shifted to the design of whole-cell biocatalysts. Besides enhancing enzyme stability, whole cells provide the advantage that the cofactor for the NADPH-dependent reductases is regenerated through the cell metabolism. For the construction of *E.* *coli* whole-cell biocatalysts, the strain C41(DE3) was chosen due to the high expression levels (and thus resulting in high activities) of AtPrR2 and FiPLR (Additional file 2), as well as PsVAO (data not shown).

**Fig. 2:**
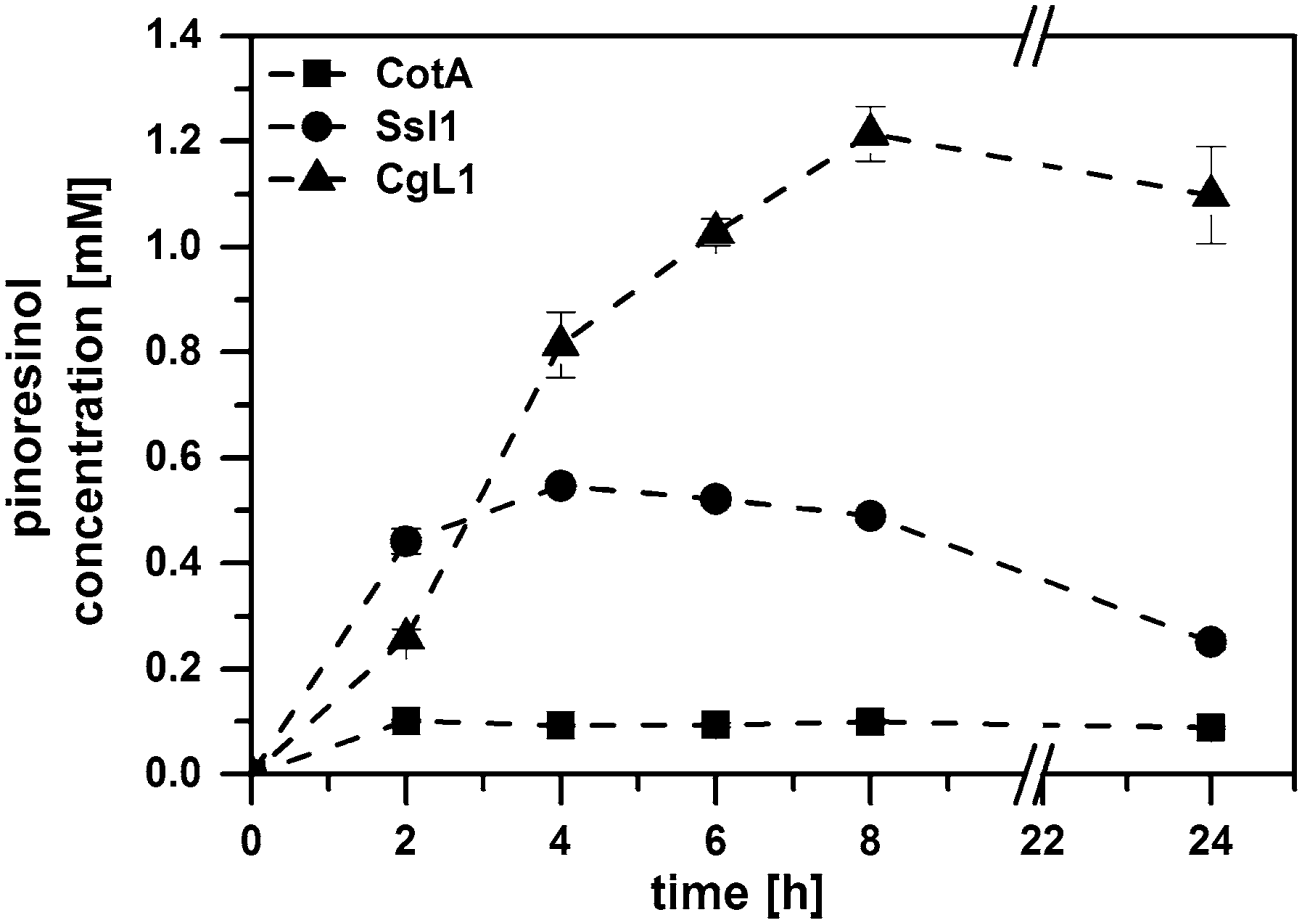
Achieved concentrations of (±)-pinoresinol **3** in whole-cell biotransformations combining PsVAO and a bacterial laccase (CotA, Ssl1, or CgL1). Reaction conditions: 10 mM eugenol **1**, 70 g l^−1^ cww *E.* *coli* C41(DE3) harbouring PsVAO and a bacterial laccase, resuspended in 50 mM KPi-buffer, pH 7.5, 0.1 mM IPTG

Our previous results demonstrated that an adjustment of PsVAO and bacterial laccase activities was essential for increasing the yield of (±)-pinoresinol **3** in vitro [17]. Therefore, in a first step, the most suitable laccase for the conversion of coniferyl alcohol **2** in the in vivo process had to be identified. Resting *E.* *coli* cells expressing PsVAO and one of the three bacterial laccases (CotA from *Bacillus licheniformis*, Ssl1 from *Streptomyces sviceus* or CgL1 from *Corynebacterium glutamicum)* were tested for the conversion of 10 mM eugenol **1** to (±)-pinoresinol **3**. For the co-expression of PsVAO and CotA almost no formation of (±)-pinoresinol **3** was observed, although eugenol **1** and the intermediate coniferyl alcohol **2** were converted completely. During co-expression of PsVAO and Ssl1 the formation of (±)-pinoresinol **3** reached 550 µM after 4 h and decreased thereafter. The highest yield of (±)-pinoresinol **3** of 1.2 mM was achieved with *E.* *coli* cells co-expressing PsVAO and CgL1 (Fig. 2). The low yields of (±)-pinoresinol **3** in the reactions containing CotA or Ssl1 can presumably be explained by the further oxidation of (±)-pinoresinol **3** by these laccases [17]. Higher expression levels of CotA (3400 mU ml^−1^) and Ssl1 (900 mU ml^−1^) compared to CgL1 (177 mU ml^−1^), as well as higher redox potentials of CotA and Ssl1 result in faster oxidation of (±)-pinoresinol **3** [17].

### In vivo one-pot “one-cell” cascade reaction for the synthesis of enantiopure (+)-pinoresinol

**Fig. 3:**
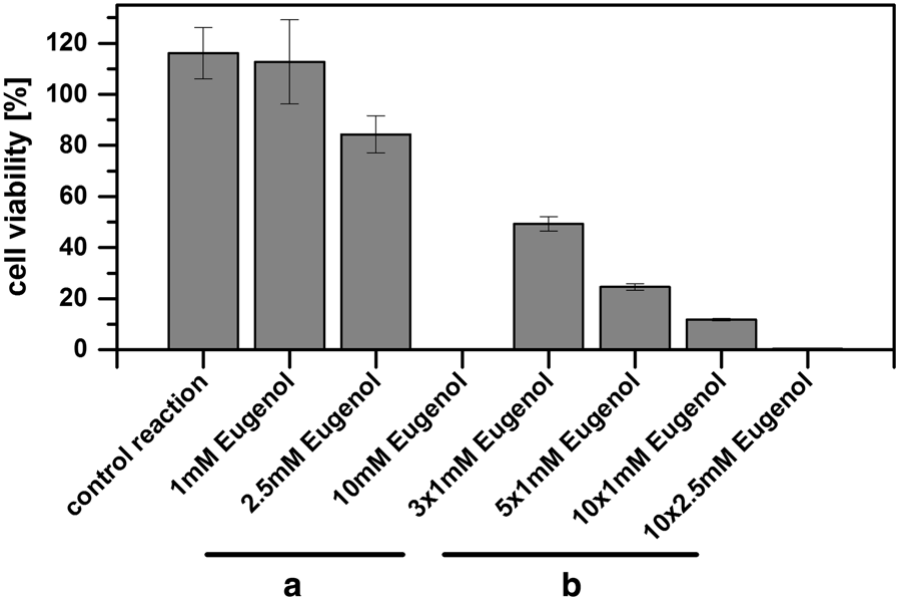
Cell viability of resting *E.* *coli* cells harbouring PsVAO, CgL1, and AtPrR2 after incubation for 24 h. The influence of different concentrations of eugenol **1** is shown. **a** Addition of eugenol **1** in one step; **b** stepwise addition of eugenol **1** as indicated. Data are plotted in relation to the amount of living cells at t = 0 h that was set as 100 %

In a first trial all enzymes (PsVAO, CgL1, and AtPrR2) were co-expressed in the *E.* *coli* strain C41(DE3) to furnish enantiopure (+)-pinoresinol **3a** by a whole-cell biocatalyst. The cells were harvested after enzyme expression, resuspended in 50 mM potassium phosphate (KPi)-buffer, pH 7.5, and supplemented with eugenol **1**. The addition of 1 mM or 2.5 mM eugenol **1** yielded (+)-pinoresinol **3a** with ee values of 74 and 88 %, respectively (Table 1, entries 1, 2), whereas with 10 mM of eugenol **1** the ee value reached only 4 % (Table 1, entry 3). In search of an explanation for the decreased ee value at a high concentration of eugenol **1**, it was found that eugenol **1** had previously been described to be toxic for *E.* *coli* [34, 35]. Indeed, cell growth analysis in the presence of different concentrations of eugenol **1** (1, 2.5, 5, or 10 mM) revealed that concentrations of eugenol **1** above 5 mM were highly toxic (no further cell growth was observed; see Additional file 3). The addition of 2.5 mM eugenol **1** reduced cell growth by about 70 %, whereas 1 mM eugenol **1** had the slightest effect on cell growth (25 % reduced OD_600_ compared to control reaction without eugenol **1**). In addition, cell viability tests with resting *E.* *coli* cells revealed that eugenol **1** concentrations of 10 mM were highly toxic and led to cell lysis (Fig. 3). Upon cell lysis AtPrR2 gets exposed to high concentrations of eugenol **1** and additionally the cofactor regeneration by the cell metabolism is no longer assured, which presumably explains the loss of its function.

**Table 1:** Concentrations of pinoresinol **3** and corresponding ee values achieved in the three-step one-pot system

Entry	Added concentration of **1** ^a^	Addition of	Concentration of **3** (µM)	ee value (%)
1	1 x 1 mM^b^	–	6 ± 5	74 [(+)-**3a**]
2	1 x 2.5 mM^b^	–	32 ± 8	88 [(+)-**3a**]
3	1 x 10 mM^b^	–	995 ± 119	4 [(+)-**3a**]
4	10 x 2.5 mM^b^	**1** every 1 h	2730 ± 10	25 [(+)-**3a**]
5	10 x 1 mM^b^	**1** every 1 h	1030 ± 70	37 [(+)-**3a**]
6	5 x 1 mM^b^	**1** every 2 h	190 ± 20	44 [(+)-**3a**]
7	3 x 1 mM^b^	**1** every 4 h	63 ± 9	≥99 [(+)-**3a**]
8	1 x 10 mM^c,d^	–	1472 ± 16	1 [(+)-**3a**]
9	1 x 10 mM^c,e^	C41_AtPrR2_	822 ± 44	97 [(+)-**3a**]
10	1 x 10 mM^c,f^	C41_AtPrR2_	876 ± 21	98 [(+)-**3a**]
11	1 x 10 mM^c,g^	C41_FiPLR_	610 ± 19	97 [(−)-**3b**]
12	1 x 10 mM^c,e^	C41_FiPLR_	456 ± 19	95 [(−)-**3b**]
13	1 x 10 mM^c,h^	C41_FiPLR_	434 ± 40	92 [(−)-**3b**]

All reaction conditions tested yielded 100 % conversion of eugenol **1**

^a^Reaction conditions: reaction buffer (50 mM KPi-buffer, pH 7.5, 100 µM IPTG), 2 % (v/v) dimethyl sulfoxide (DMSO), concentrations of eugenol **1** as indicated. Reactions were carried out for 24 h at 25 °C, 140 rpm

^b^Recombinant *E.* *coli* C41(DE3) harbouring PsVAO, CgL1, and AtPrR2 resuspended in 10 ml reaction buffer with an adjusted cell wet weight (cww) of 70 g l^−1^

^c^Recombinant *E.* *coli* C41(DE3) harbouring PsVAO and CgL1 resuspended in 10 ml reaction buffer with 20 g l^−1^ d-glucose (cww adjusted to 70 g l^−1^)

^d^Without addition of recombinant *E.* *coli* cells harbouring AtPrR2 or FiPLR

^e^Addition of recombinant *E.* *coli* cells harbouring AtPrR2 or FiPLR resuspended in 10 ml reaction buffer with 20 g l^−1^ d-glucose (cww adjusted to 70 g l^−1^) after 24 h; further incubation for 4 h

^f^As reaction e, but further incubation for 8 h

^g^As reaction e, but further incubation for 2 h

^h^As reaction e, but further incubation for 6 h

**Fig. 4:**
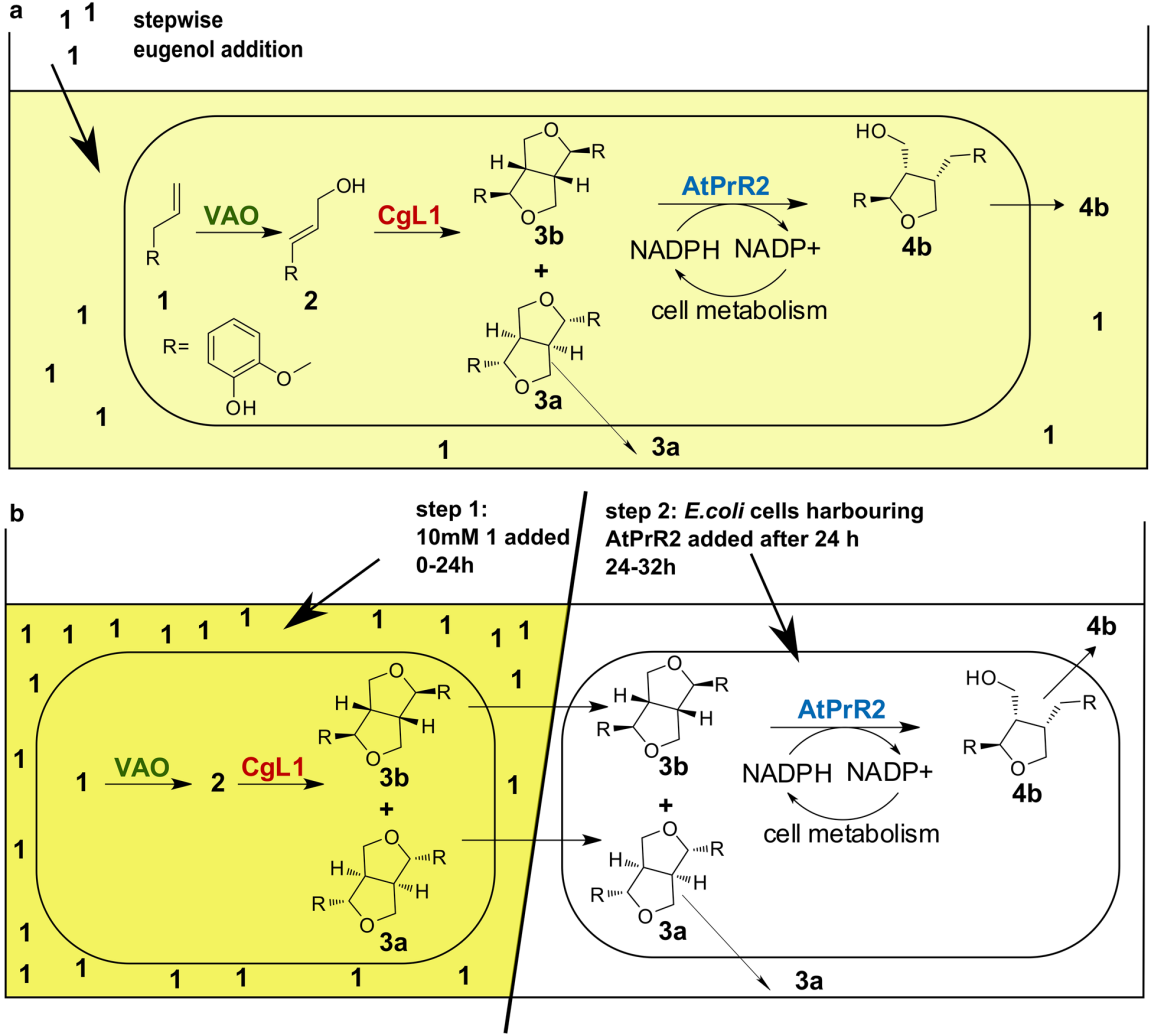
Two optimization approaches for the synthesis of enantiopure pinoresinol **3**. **a** Stepwise addition of 1 or 2.5 mM eugenol **1** to resting *E.* *coli* cells harbouring PsVAO, CgL1, and AtPrR2; **b** addition of 10 mM eugenol **1** in a one-pot “two-cell” sequential mode of operation: The substrate was added to resting *E.* *coli* cells harbouring PsVAO and CgL1. After 24 h resting *E.* *coli* cells harbouring AtPrR2 were added. Intensity of *yellow colour* indicates eugenol **1** concentration in the reaction

To circumvent the limitation of substrate toxicity, a step-wise addition of eugenol **1** was applied to the “one-cell” system with resting *E.* *coli* cells harbouring all three enzymes (PsVAO, CgL1, and AtPrR2) (Fig. 4a). The cells were first supplemented with low concentrations of eugenol **1** (1 or 2.5 mM) and incubated for 1 h. After that, doses of 1 or 2.5 mM of eugenol **1** were added every 1 h for a time period of 9 h. Compared to the initial experiments with 10 mM of eugenol **1** added at once, the ee value could be increased up to 37 % (Table 1, entries 4, 5). A slower addition of 1 mM eugenol **1** every 2 h (Table 1, entry 6) or every 4 h (Table 1, entry 7) resulted in ee values of 44  and ≥99 %, respectively, but the concentrations of (+)-pinoresinol **3a** achieved were lower (190 and 63 µM, respectively). In all cases, the obtained ee values were in accordance with the observed cell viability; they were increasing with increasing viability of the cells, which points out the importance that intact cells are required in order to achieve high AtPrR2 activity.

We observed that during the course of the reaction, formation of a side product occured that we identified as coniferyl aldehyde **6**. This side reaction could not be attributed to any of the enzymes of the cascade. To understand the origin of coniferyl aldehyde **6**, control reactions were performed with resting *E.* *coli* cells that did not express heterologous enzymes but were supplemented with eugenol **1**, coniferyl alcohol **2**, or pinoresinol **3**. While no conversion of eugenol **1** and pinoresinol **3** was seen, coniferyl alcohol **2** was oxidized by *the E.* *coli* cells to coniferyl aldehyde **6**. Presumably, coniferyl alcohol **2** is used to regenerate NADPH due to a shifted NADPH/NADP^+^ equilibrium towards NADP^+^ within the cell. To prove this hypothesis, either 25 g l^−1^ glycerol or 20 g l^−1^d-glucose were added to *E.* *coli* cells harbouring PsVAO and CgL1 as an energy source to ensure higher NADPH concentrations. As expected, conversion of 2.5 mM eugenol **1** with addition of glycerol or d-glucose resulted in higher concentrations of (±)-pinoresinol **3** (351 and 375 µM, compared to 153 µM; Table 2) and reduced coniferyl aldehyde **6** formation (see Additional file 4).

**Table 2:** Achieved concentration of pinoresinol **3** and ee values for (+)-**3a** in the one-pot “one-cell” set-up depending on the energy source added

*E.* *coli* cell	Energy source	Concentration of **3** (µM)	ee value of ±**3a** (%)
C41(DE3) harbouring PsVAO and CgL1	None added	153 ± 40.7	4
	25 g l^−1^ glycerol	351 ± 27	8
	20 g l^−1^ d-glucose	375 ± 17	5
C41(DE3) harbouring PsVAO and CgL1 and AtPrR2	None added	42 ± 3	≥99
	25 g l^−1^ glycerol	308 ± 29	2
	20 g l^−1^ d-glucose	402 ± 28	0

All reactions yielded 100 % conversion of eugenol **1**

Reaction conditions: recombinant *E.* *coli* cells resuspended in 10 ml 50 mM KPi-buffer, pH 7.5, 100 µM IPTG (cww = 70 g l^−1^); 2 % (v/v) DMSO; 2.5 mM of eugenol **1**. Reactions were carried out for 24 h at 25 °C, 140 rpm

When the same reaction set-up was assigned to *E.* *coli* cells harbouring PsVAO, CgL1, and AtPrR2, similar amounts of (±)-pinoresinol **3** were detected (308 and 402 µM), but unexpectedly no conversion of (−)-pinoresinol **3b** to (−)-lariciresinol **4b** was obtained (ee % = 2 and 0; Table 2). We speculate that due to reduced formation of coniferyl aldehyde **6** under these conditions the accumulation of higher concentrations of coniferyl alcohol **2** could either be toxic for the cells and/or negatively affect the activity of AtPrR2 (as was observed for eugenol **1**), but this was not investigated in detail.

### In vivo one-pot “two-cell” sequential cascade reaction for the synthesis of enantiopure (+)-pinoresinol

Because of the correlation between cell viability and AtPrR2 activity, and the observation that higher concentrations of (±)-pinoresinol **3** were achieved in the presence of d-glucose, we decided to separate the kinetic resolution step of (±)-pinoresinol **3** from its production step. The one-pot approach was set up in a sequential mode of operation (Fig. 4b) as follows: High concentrations of eugenol **1** were added to a first set of resting *E.* *coli* cells harbouring PsVAO and CgL1, but not AtPrR2. The first two biocatalytic steps of this cascade are independent from cofactors, and PsVAO and CgL1 are stable in the presence of high concentrations of eugenol **1**; therefore cell lysis due to substrate toxicity does not affect the production of (±)-pinoresinol **3**. After 24 h freshly prepared resting *E.* *coli* cells harbouring AtPrR2 were added to the reaction and continued for additional 4–8 h. Under these conditions, at 10 mM of eugenol **1**, up to 876 µM (+)-pinoresinol **3a** with an ee value of 98 % were gained (Table 1, entries 9, 10). The maximal theoretical molar yield of enantiopure pinoresinol **3** starting from eugenol **1** is 25 %. In comparison, we achieved 8.8 % which corresponds to one third of the maximal yield. This difference can be explained through side product formation due to the radical reaction mechanism of laccases, as described previously [17, 23]. As a consequence, 1472 µM (±)-pinoresinol **3** were achieved starting from 10 mM eugenol **1** at almost complete conversion of substrate **1** and intermediate **2**.

Encouraged by these results an upscaling experiment was performed: 160 mg eugenol **1** (10 mM, 0.98 mmol) were added to 100 ml resting *E.* *coli* cells harbouring PsVAO and CgL1 (resuspended in reaction buffer; see “Methods” section). After 24 h 100 ml resting *E.* *coli* cells harbouring AtPrR2 were added and the reaction was continued for additional 4 h. Enantiopure (+)-pinoresinol **3a** (see Additional file 5A) and (−)-lariciresinol **4b** (which is not commercially available) were purified from the reaction, and isolated yields of 12 % (19 mg) and 11 % (18 mg) were achieved, respectively.

### In vivo one-pot “two-cell” sequential cascade reaction for the synthesis of enantiopure (−)-pinoresinol

Besides the production of (+)-pinoresinol **3a**, we investigated the potential of the one-pot “two-cell” system for the production of enantiopure (−)-pinoresinol **3b**. This compound is not commercially available and its effective production is particularly attractive. *E.* *coli* cells harbouring FiPLR from *F.* *intermedia* with an opposite enantioselectivity to AtPrR2 were applied for the kinetic resolution step. Utilizing this set-up under the established reaction conditions, the concentrations of (−)-pinoresinol **3b** achieved 610 µM with an ee value of 97 % (Table 1, entries 11–13), which is in a similar range compared to the system producing (+)-pinoresinol **3a**. Moreover, the high-value compound (−)-secoisolariciresinol **5a** was formed with an ee value of ≥99 % through the further oxidation of (+)-lariciresinol **4a** by FiPLR (see Additional file 5B).

## Conclusions

Within this study, we demonstrated that the three-step cascade including a kinetic resolution step is a powerful approach for the synthesis of enantiopure pinoresinol **3** starting from the inexpensive substrate eugenol **1**. A sequentially operating one-pot “two-cell” process is preferable to a simultaneous one-pot “one-cell” mode of operation. It was demonstrated that the process could easily be switched from production of enantiopure (+)-pinoresinol **3a** to enantiopure (−)-pinoresinol **3b** by choosing a plant reductase with opposite enantioselectivity.

In addition, the high-value compound (−)-lariciresinol **4b** was isolated in enantiopure form, and the formation of enantiopure (−)-secoisolariciresinol **5a** was demonstrated. This enables the development of biocatalytic systems for the production of lariciresinol **4** and secoisolariciresinol **5** that can serve as building blocks for the production of other lignans.

## Methods

### Enzymes and chemicals

Pinoresinol **3** (≥95 %, SML0073; mixture composed of 61 % **3a** and 39 % **3b**), (+)-lariciresinol **4a** (≥95 %, 06892), and secoisolariciresinol **5** (≥95 %, 60,372) were obtained in HPLC grade from Sigma-Aldrich. All other chemicals were purchased in an analytical or higher grade from Sigma-Aldrich, Alfa Aesar, or Merck. LC/MS grade solvents were from Sigma-Aldrich (water) and Fisher Scientific (formic acid and methanol). HPLC grade solvents were obtained from Carl Roth (*n*-heptane), Th. Geyer (*n*-hexane), and Sigma-Aldrich (ethanol). Enzymes for molecular biology (DNA-polymerase, restriction endonucleases, T4-DNA-ligase) were acquired from Thermo Scientific. “Anti-His_6_-Peroxidase (2)” and the “BM Blue POD Substrate (precipitating)” from Roche were used for Western-Blot analysis.

### Synthetic genes and molecular biology

#### Synthetic genes

Synthetic genes *syatprr2* and *syfiplr* (see Additional file 1) were ordered codon optimized for *E.* *coli* from Eurofins MWG Operon. A C-terminal hexa-histidine tag (His_6_-tag) was added to the sequence of *syfiplr*.

#### Cloning of reductase genes

The genes *syatprr2* and *syfiplr* were amplified by polymerase chain reaction (PCR) using the synthetic genes as template and the oligonucleotide sequences (Eurofins MWG Operon) listed in Table 3. The restrictions sites of the endonucleases *Nde*I and *Xho*I are underlined, and the His_6_-tag sequence is marked in bold.

**Table 3:** Oligonucleotides used for polymerase chain reaction

Gene	Oligonucleotide	
	Name	Sequence
*syatprr*2 (with His_6_-tag)	syatprr2_tag_fw	5′-GGGTTTCATATGAAAGAGACTAACTTCGGCG-3′
	syatprr2_tag_rev	5′-CCGCTCGAGTCA**GTGGTGATGATGGTGATG**ACCGCCGACGAAAATTTTCAG-3′
*syfiplr (*with His_6_-tag)	syfiplr_tag_fw	5′-GGGTTTCATATGGGCAAATCCAAAGTTCTG-3′
	syfiplr_tag_rev	5′-CCGCTCGAGTCAGTGGTGATGATGG-3′
*syfiplr* (no tag)	syfiplr_fw	5′-GGGTTTCATATGGGCAAATCCAAAGTTCTG-3′
	syfiplr_rev	5′-CCGCTCGAGTCAAACATAGCGTTTAAGGTATTCTTCAAC-3′

The amplified DNA fragments and the plasmid pCDF-Duet were cut with *Nde*I and *Xho*I and ligated by T4-DNA-ligase resulting in the expression plasmids pCDF-Duet_syatprr2_his_6_, pCDF-Duet_syfiplr_his_6_, and pCDF-Duet_syfiplr. Correct insertion was verified by Sanger DNA sequencing (GATC Biotech).

### Cloning of *psvao* and co-expression plasmids

For whole-cell biotransformations different plasmids for the co-expression of certain genes were produced (see Additional file 1): (1) pACYC_tac__psvao, (2) pCDF-Duet_psvao_syatprr2_his_6_, (3) pCDF-Duet_psvao_syfiplr.

First, the gene *psvao* was inserted into the plasmid pACYC_tac_ (kindly provided by Dr. Natalie Trachtmann, Institute of Microbiology, University of Stuttgart). Amplification by PCR took place using pET28b_psvao [17] as template and the oligonucleotides 5′-AACGAGCTCGATGTCCAAGACACAGG-3′ and 5′-CCCAAGCTTGGGTTACAGTTTCC-3′. Restriction of the amplified DNA fragments and pACYC_tac_ was done with the restriction endonucleases *Sac*I and *Hin*dIII.

Next, *psvao* was also cloned upstream of reductase genes into the first multiple cloning site (MCS) of the pCDF-Duet-based expression plasmids generated before. *Psvao* was cut out of pACYC_tac__psvao with *Sac*I and *Hind*III and ligated into the plasmids pCDF-Duet_syatprr2_his_6_ or pCDF-Duet_syfiplr. After ligation the resulting plasmids were confirmed by Sanger DNA sequencing (GATC Biotech).

### Heterologous expression in *E.* *coli*

For heterologous expression of genes (*syatprr*2, *syfiplr*, *psvao)* different *E.* *coli* strains were tested: BL21(DE3), OverExpress C41(DE3), OverExpress C43(DE3), and Shuffle T7 Express. 5 ml LB medium supplemented with 50 µg ml^−1^ streptomycin (*syatprr*2, *syfiplr*), or 34 µg ml^−1^ chloramphenicol (*psvao*), were inoculated with a single colony and incubated over night at 37 °C, 180 rpm. Expression was performed in 50 ml TB-medium with the corresponding antibiotic inoculated with 500 µl of the pre-culture and incubated at 37 °C, 180 rpm to an optical density at 600 nm (OD_600_) of 0.6. 0.5 mM isopropyl β-D-1-thiogalactopyranoside (IPTG) was added to the culture and thereafter incubated at 30 °C, 140 rpm for 21 h. The culture was harvested by centrifugation at 3200×*g* and resuspended in 5 ml 50 mM KPi-buffer, pH 7.5 with 0.1 mM phenylmethylsulfonyl fluoride (PMSF). Cell lysis was performed by sonication on ice, and cell debris was removed by centrifugation at 11,325×*g* for 20 min.

Expressions of the laccase gene *cgl*1 from *Corynebacterium glutamicum* [36] and the glucose dehydrogenase (GDH) gene *gdh*IV from *Bacillus megaterium* [37] were performed as described previously.

### Determination of enzymatic activities

#### Activity assay for reductases

The activities of the soluble protein fractions of reductase expressions were analysed towards pinoresinol **3** in a reaction volume of 500 µl. 50 µl soluble protein fraction was added to 200 µM pinoresinol **3** and 200 µM NADPH in 50 mM KPi-buffer, pH 7.5 and incubated at 25 °C for 16 h. Optionally, a cofactor regeneration system consisting of 20 mM d-glucose and 3 U ml^−1^ GDH was added.

For LC/MS analysis 100 µM ferulic acid methyl ester [FSME; 5 mM stock solution in dimethyl sulfoxide (DMSO)] was added as internal standard and the reaction was extracted with 600 µl ethyl acetate. The organic phase was evaporated and the residue was resuspended in 100 µl methanol. Non-chiral LC/MS analysis was performed as described below.

The influence of eugenol **1** and *t*BME on the reductase activity was tested as follows: eugenol **1** (1, 2.5, or 10 mM) or 20 % (v/v) *t*BME were added to the established activity assay, and the reactions were performed and analysed by non-chiral LC/MS as described.

#### Activity assays for PsVAO and CgL1

The activities of PsVAO and CgL1 were determined as described previously [17].

### In vitro one-pot three-step cascade reaction for the synthesis of enantiopure pinoresinol

The reaction for the synthesis of enantiopure (+)-pinoresinol **3a** was set up by addition of the reductase AtPrR2 to the previously established in vitro cascade reaction [17]. 1 mM eugenol **1** in 50 mM KPi-buffer, pH 7.5 was supplemented with 20 % (v/v) *t*BME, 10 mU ml^−1^ PsVAO, 50 mU ml^−1^ CgL1, 0.03 mU ml^−1^ AtPrR2, 200 µM NADPH, 20 mM d-glucose, and 3 U ml^−1^ GDH. Samples were incubated at 25 °C for 7.5 h in an overhead shaker (20 rpm).

For GC/MS analysis 100 µM FSME was added, and extraction was performed twice with 300 µl ethyl acetate. All samples were analysed by GC/MS and non-chiral LC/MS as described below.

### Laccase screening

For whole-cell biotransformations of eugenol **1** combinations of PsVAO and different bacterial laccases (CotA, Ssl1, or CgL1) were analysed. *E.* *coli* OverExpress C41(DE3) was chosen due to high expression levels of the recombinant enzymes.

Competent C41(DE3) cells were co-transformed with the following plasmids: (1) pACYC_tac__psvao and pETK316 N/D500G, (2) pACYC_tac__psvao and pET22ssl1, or (3) pACYC_tac__psvao and pET16b_cgl1 (see Additional file 1). Expressions were carried out in 200 ml TB-medium supplemented with 100 µg ml^−1^ ampicillin and 34 µg ml^−1^ chloramphenicol at 37 °C, 180 rpm. At an OD_600_ of 0.6 0.5 mM IPTG and CuSO_4_ (2 mM in case of CotA and Ssl1, 3 mM in case of CgL1) were added. Thereafter, cultures were incubated at 30 °C, 140 rpm for 21 h.

Cells were harvested by centrifugation and resuspended in reaction buffer (50 mM KPi, pH 7.5; 100 µM IPTG) thereby adjusting a cell wet weight of 70 g l^−1^ (corresponding to 18.1 g l^−1^ cell dry weight). 10 ml of resuspended cells were exposed to 10 mM eugenol **1** and 2 % (v/v) DMSO and incubated at 25 °C, 140 rpm in an orbital shaker for 24 h. Samples (0.5 ml) were taken after certain time points, 2 mM FSME (100 mM stock solution in DMSO) was added and extracted with 1 ml ethyl acetate. All samples were analysed by GC/MS as described below.

### In vivo one-pot “one-cell” cascade reaction for synthesis of enantiopure pinoresinol

*E.* *coli* OverExpress C41(DE3) cells co-expressing the plasmids pCDF-Duet_psvao_systprr2_his_6_ and pET16b_cgl1 were employed. Protein expression and adjustment of cell wet weight was conducted as described above for the co-expression of PsVAO and a bacterial laccase.

The one-pot “one-cell” cascade was analysed regarding (1) different eugenol **1** concentrations (1–10 mM), (2) addition of different energy sources (20 g l^−1^d-glucose or 25 g l^−1^ glycerol), and (3) the stepwise addition of eugenol **1** (1 mM or 2.5 mM added as indicated in Table 1).

Control reactions were performed in the same manner but with *E.* *coli* cells not expressing heterologous genes; 1 mM eugenol **1**, 1 mM coniferyl alcohol **2**, or 0.75 mM pinoresinol **3** were added to the cells.

Extraction was performed with 6 ml ethyl acetate after addition of 100 µM FSME (for concentrations of eugenol **1** of up to 3 mM) or 2 mM FMSE (in all other cases). All samples were analysed by CG/MS, non-chiral LC/MS, and chiral HPLC as described below.

### In vivo one-pot “two-cell” sequential cascade reactions for synthesis of enantiopure pinoresinol

The one-pot “two-cell” sequential reaction was realized by employing two types of cells: Conversion of eugenol **1** to (±)-pinoresinol **3** was achieved by *E.* *coli* OverExpress C41(DE3) cells carrying plasmids pACYC_tac__psvao and pET16b_cgl1, whereas kinetic resolution of (±)-pinoresinol **3** was done by *E.* *coli* OverExpress C41(DE3) cells carrying either pCDF-Duet_syatprr2_his_6_ (for synthesis of (+)-pinoresinol **3a**), or pCDF-Duet_syfiplr (for synthesis of (−)-pinoresinol **3b**). Protein expression was conducted as described above for the co-expression of PsVAO and a bacterial laccase.

The first step of the reaction was performed with resting *E.* *coli* cells with heterologously expressed PsVAO and CgL1 (resuspended in 10 ml reaction buffer supplemented with 25 g l^−1^d-glucose; cell wet weight adjusted to 70 g l^−1^), 2 % (v/v) DMSO, 10 mM eugenol **1**. After 24 h resting *E.* *coli* cells with heterologously expressed AtPrR2 or FiPLR were added (resuspended in 10 ml reaction buffer supplemented with 25 g l^−1^d-glucose; cell wet weight adjusted to 70 g l^−1^). Extraction was performed with 6 ml ethyl acetate after addition of 2 mM FMSE. All samples were analysed by GC/MS, non-chiral LC/MS, and chiral HPLC as described below.

For scale-up experiments, the reaction was multiplied by the factor of 10 resulting in a substrate amount of 160 mg (10 mM in 100 ml), 100 ml *E.* *coli* cells harbouring PsVAO and CgL1, and 100 ml *E.* *coli* cells harbouring AtPrR2.

Pinoresinol **3** and lariciresinol **4** were purified from the reaction mixture by preparative HPLC. For product verification high-resolution mass spectrometry (HR/MS), ^1^H NMR, ^13^C NMR, HMBC, COSY, and HSQC were performed (for additional information and results see Additional file 6).

### Cell toxicity tests

#### Cell growth assay

The effect of different eugenol **1** concentrations on cell growth of *E.* *coli* OverExpress C41(DE3) cells carrying pACYC_tac__psvao and pET16b_cgl1 was monitored. 400 ml TB-medium were inoculated with 4 ml of an overnight culture and incubated at 37 °C, 180 rpm to an OD_600_ of 0.6. Cells were harvested by centrifugation, resuspended in TB-medium adjusting an OD_600_ = 0.6, and split. Different concentrations of eugenol **1** (0, 1, 2.5, 5, or 10 mM) and 2 % (v/v) DMSO were added. Cells were incubated at 37 °C, 180 rpm and cell growth was analysed for additional 18 h by measuring the OD_600_.

#### Cell viability assay

Viability of resting *E.* *coli* cells during biotransformations was assayed as follows: 40 µl of the cell suspension was withdrawn immediately after eugenol **1** addition, as well as after 24 h reaction time. The samples were diluted and plated on LB-agar-plates containing 100 µg ml^−1^ ampicillin and 50 µg ml^−1^ streptomycin. After incubation at 37 °C over night colony forming units (CFU) were counted.

### Reaction analysis by GC/MS, LC/MS, and HPLC

GC/MS analysis was performed as described previously [17].

Non-chiral LC/MS measurements were performed on a LC/MS-2020 (Shimadzu, Duisburg, Germany) equipped with a Chromolith^®^ Performance RP-18e column (100 × 4.6 mm, Merck, Darmstadt, Germany). A solvent gradient of methanol and 0.1 % formic acid at a flow rate of 0.5 ml min^−1^ was applied as follows: starting from 20 to 35 % methanol in 5 min, hold for 5 min, increase to 70 % methanol within 15 min, then to 90 % methanol within 1 s, hold for 1 min, re-equilibration with 20 % methanol. UV/Vis spectra were monitored in the range between 190–800 nm. The interface temperature was 350 °C, the desolvation line temperature was 275 °C, and the heat block temperature was 400 °C. The nebulizing gas flow and the drying gas flow were set to 1.5 and 15 l min^−1^, respectively.

For determination of the enantiomeric composition of pinoresinol **3**, lariciresinol **4**, and secoisolariciresinol **5** reaction mixtures were analysed by chiral HPLC (Shimadzu, Duisburg, Germany) equipped with a CHIRALPAK^®^IB column (250 × 4.6 mm, Chiral Technologies Europe, Illkirch Cedex, France). The solvents *n*-hexane/ethanol were used under isocratic conditions (pinoresinol **3**: 50/50; lariciresinol **4**: 80/20, secoisolariciresinol **5**: 75/25) at a flow rate of 0.7 ml min^−1^.

### Product isolation and identification

Pinoresinol **3** and lariciresinol **4** were purified by preparative HPLC equipped with a MultoHigh 100 Si-10 µ column (250 × 10 mm, pore size 100 Å, 10 µm particle size, CS-Chromatographie Service, Langerwehe, Germany). A solvent gradient of *n*-heptane and ethanol at a flow rate of 7.5 ml min^−1^ was applied as follows: starting from 10 to 39 % ethanol in 12.5 min, increase to 90 % ethanol within 1 s, hold for 1 min, re-equilibration with 10 % ethanol.

Product identification was performed by NMR and HR/MS as described in the Additional file 6.

## Additional files

10.1186/s12934-016-0472-0 DNA-sequences of the synthetic genes (*syatprr*2 and *syfiplr*), plasmids and strains used within this study.
10.1186/s12934-016-0472-0 Formation of lariciresinol **4** or secoisolariciresinol **5** from pinoresinol **3** in the reactions with cleared cell lysates of different *E.* *coli* strains expressing the reductase AtPrR2 (**a**) or the reductase FiPLR (**b**). Reaction conditions: 50 mM KPi-buffer, pH 7.5, 0.2 mM pinoresinol **3**, 50 µl cleared cell lysate containing AtPrR2 or FiPLR, 0.2 mM NADPH. Optionally, 3 U ml^−1^ GDH and 20 mM D-glucose were added for cofactor regeneration (cr). The reactions were incubated at 25 °C for 16 h. Besides formation of lariciresinol **4** or secoisolariciresinol **5**, no other reaction products were observed.
10.1186/s12934-016-0472-0 Cell growth of *E.* *coli* C41(DE3) cells co-transformed with pACYC_tac__psvao and pET16b_cgl1 in the presence of different concentrations of eugenol **1**. Cell cultures were grown at 37 °C, 180 rpm to an OD_600_ of 0.6 and harvested (indicated by an arrow). Cell pellet was resuspended in TB medium, eugenol **1** was added and the cultures were incubated at 37 °C, 180 rpm. Cell growth was monitored at 600 nm. Black square: 0 mM eugenol **1**, blue circle: 1 mM, green triangle: 2.5 mM, purple square: 5 mM, orange circle: 10 mM.
10.1186/s12934-016-0472-0 Influence of an energy source on conversion of coniferyl alcohol **2** by *E.* *coli* cells. 10 ml *E.* *coli* C41(DE3) pCDF-Duet resuspended in reaction buffer (70 g l^−1^ cww) were supplemented with 1 mM coniferyl alcohol **2**. Optionally, 25 g l^−1^ glycerol or 20 g l^−1^ D-glucose was added. The reaction was carried out for 24 h at 25 °C, 140 rpm. [2] = coniferyl alcohol **2**, [6] = coniferyl aldehyde **6**, [IS] = internal standard FSME, * = unspecific peak; chromatograms are shifted vertically.
10.1186/s12934-016-0472-0 HPLC chromatograms of enantiomeric separations of reaction products. **a** Application of AtPrR2; **b** application of FiPLR. [3a] = (+)-pinoresinol **3a**, [3b] = (−)-pinoresinol **3b**, [4a] = (+)-lariciresinol **4a**, [4b] = (−)-lariciresinol **4b**, [5a] = (−)-secoisolariciresinol **5a**.
10.1186/s12934-016-0472-0 Product identification by NMR and HR/MS.

## References

[1] Sepporta MV, Mazza T, Morozzi G, Fabiani R (2013). Pinoresinol inhibits proliferation and induces differentiation on human HL60 leukemia cells. Nutr Cancer.

[2] Menendez JA, Vazquez-Martin A, Garcia-Villalba R, Carrasco-Pancorbo A, Oliveras-Ferraros C, Fernandez-Gutierrez A, Segura-Carretero A (2008). tabAnti-HER2 (*erb*B-2) oncogene effects of phenolic compounds directly isolated from commercial Extra-Virgin Olive Oil (EVOO). BMC Cancer.

[3] Mitsuhashi S, Kishimoto T, Uraki Y, Okamoto T, Ubukata M (2008). Low molecular weight lignin suppresses activation of NF-kappaB and HIV-1 promoter. Bioorg Med Chem.

[4] Wikul A, Damsud T, Kataoka K, Phuwapraisirisan P (2012). (+)-Pinoresinol is a putative hypoglycemic agent in defatted sesame (*Sesamum indicum*) seeds though inhibiting α-glucosidase. Bioorg Med Chem Lett.

[5] Kim KH, Moon E, Kim SY, Lee KR (2010). Lignans from the tuber-barks of *Colocasia antiquorum* var. *esculenta* and their antimelanogenic activity. J Agric Food Chem.

[6] Lapi D, Di Maro M, Mastantuono T, Battiloro L, Sabatino L, Muscariello E, Colantuoni A (2015). Effects of oleuropein and pinoresinol on microvascular damage induced by hypoperfusion and reperfusion in rat pial circulation. Microcirculation.

[7] Hwang B, Lee J, Liu Q, Woo E, Lee DG (2010). Antifungal effect of (+)-pinoresinol isolated from *Sambucus williamsii*. Molecules.

[8] Landete JM (2012). Plant and mammalian lignans: a review of source, intake, metabolism, intestinal bacteria and health. Food Res Int.

[9] Feng J, Shi Z, Ye Z (2008). Effects of metabolites of the lignans enterolactone and enterodiol on osteoblastic differentiation of MG-63 cells. Biol Pharm Bull.

[10] McCann MJ, Rowland IR, Roy NC (2014). The anti-proliferative effects of enterolactone in prostate cancer cells: evidence for the role of DNA licencing genes, mi-R106b cluster expression, and PTEN dosage. Nutrients.

[11] Kulik T, Buśko M, Pszczółkowska A, Perkowski J, Okorski A (2014). Plant lignans inhibit growth and trichothecene biosynthesis in *Fusarium graminearum*. Lett Appl Microbiol.

[12] Grougnet R, Magiatis P, Mitaku S, Terzis A, Tillequin F, Skaltsounis A (2006). New lignans from the perisperm of *Sesamum indicum*. J Agric Food Chem.

[13] Kitagawa S, Hisada S, Nishibe S (1984). Phenolic compounds from *Forsythia* leaves. Phytochemistry.

[14] Okunishi T, Umezawa T, Shimada M (2001). Isolation and enzymatic formation of lignans of *Daphne genkwa* and *Daphne odora*. J Wood Sci..

[15] Maruyama J, Kobayashi M, Miyashita M, Kouno I, Irie H (1994). A synthesis of (±)-pinoresinol and its related compound using potassium persulfate (K2S2O8) oxidation of benzoylacetates. Heterocycles.

[16] Vermes B, Seligmann O, Wagner H (1991). Synthesis of biologically active tetrahydro-furofuranlignan-(syringin, pinoresinol)- mono- and bis-glucosides. Phytochemistry.

[17] Ricklefs E, Girhard M, Koschorreck K, Smit M, Urlacher VB (2015). Two-step one-pot production of pinoresinol from eugenol via enzymatic cascade. ChemCatChem.

[18] Davin LB, Wang HB, Crowell AL, Bedgar DL, Martin DM, Sarkanen S, Lewis NG (1997). Stereoselective bimolecular phenoxy radical coupling by an auxiliary (dirigent) protein without an active center. Science.

[19] Kim K, Moinuddin SGA, Atwell KM, Costa MA, Davin LB, Lewis NG (2012). Opposite stereoselectivities of dirigent proteins in *Arabidopsis* and *Schizandra* species. J Biol Chem.

[20] Pickel B, Schaller A (2013). Dirigent proteins: molecular characteristics and potential biotechnological applications. Appl Microbiol Biotechnol.

[21] Kazenwadel C, Klebensberger J, Richter S, Pfannstiel J, Gerken U, Pickel B (2013). Optimized expression of the dirigent protein AtDIR6 in *Pichia pastoris* and impact of glycosylation on protein structure and function. Appl Microbiol Biotechnol.

[22] Kim MK, Jeon J, Fujita M, Davin LB, Lewis NG (2002). The western red cedar (*Thuja plicata*) 8-8′ DIRIGENT family displays diverse expression patterns and conserved monolignol coupling specificity. Plant Mol Biol.

[23] Pickel B, Constantin M, Pfannstiel J, Conrad J, Beifuss U, Schaller A (2010). An enantiocomplementary dirigent protein for the enantioselective laccase-catalyzed oxidative coupling of phenols. Angew Chem Int Ed Engl.

[24] Halls SC, Lewis NG (2002). Secondary and quaternary structures of the (+)-pinoresinol-forming dirigent protein. Biochemistry.

[25] Liu J, Stipanovic RD, Bell AA, Puckhaber LS, Magill CW (2008). Stereoselective coupling of hemigossypol to form (+)-gossypol in moco cotton is mediated by a dirigent protein. Phytochemistry.

[26] Halls SC, Davin LB, Kramer DM, Lewis NG (2004). Kinetic study of coniferyl alcohol radical binding to the (+)-pinoresinol forming dirigent protein. Biochemistry.

[27] Dinkova-Kostova AT, Gang DR, Davin LB, Bedgar DL, Chu A, Lewis NG (1996). (+)-Pinoresinol/(+)-lariciresinol reductase from *Forsythia intermedia*. Protein purification, cDNA cloning, heterologous expression and comparison to isoflavone reductase. J Biol Chem.

[28] Nakatsubo T, Mizutani M, Suzuki S, Hattori T, Umezawa T (2008). Characterization of *Arabidopsis thaliana* pinoresinol reductase, a new type of enzyme involved in lignan biosynthesis. J Biol Chem.

[29] Fujita M, Gang DR, Davin LB, Lewis NG (1999). Recombinant pinoresinol-lariciresinol reductases from western red cedar (*Thuja plicata*) catalyze opposite enantiospecific conversions. J Biol Chem.

[30] Hemmati S, Schmidt TJ, Fuss E (2007). (+)-Pinoresinol/(-)-lariciresinol reductase from *Linum perenne* Himmelszelt involved in the biosynthesis of justicidin B. FEBS Lett.

[31] Wankhede DP, Biswas DK, Rajkumar S, Sinha AK (2013). Expressed sequence tags and molecular cloning and characterization of gene encoding pinoresinol/lariciresinol reductase from *Podophyllum hexandrum*. Protoplasma..

[32] Hano C, Martin I, Fliniaux O, Legrand B, Gutierrez L, Arroo RRJ (2006). Pinoresinol-lariciresinol reductase gene expression and secoisolariciresinol diglucoside accumulation in developing flax (*Linum usitatissimum*) seeds. Planta.

[33] Fukuhara Y, Kamimura N, Nakajima M, Hishiyama S, Hara H, Kasai D (2013). Discovery of pinoresinol reductase genes in sphingomonads. Enzyme Microb Technol.

[34] Overhage J, Steinbüchel A, Priefert H (2003). Highly efficient biotransformation of eugenol to ferulic acid and further conversion to vanillin in recombinant strains of *Escherichia coli*. Appl Environ Microbiol.

[35] Friedman M, Henika PR, Mandrell RE (2002). Bactericidal activities of plant essential oils and some of their isolated constituents against *Campylobacter jejuni, Escherichia coli, Listeria monocytogenes*, and *Salmonella enterica*. J Food Prot.

[36] Ricklefs E, Winkler N, Koschorreck K, Urlacher VB (2014). Expanding the laccase-toolbox: a laccase from *Corynebacterium glutamicum* with phenol coupling and cuprous oxidase activity. J Biotechnol.

[37] Khatri Y, Hannemann F, Girhard M, Kappl R, Hutter M, Urlacher VB, Bernhardt R (2015). A natural heme-signature variant of CYP267A1 from *Sorangium cellulosum* So ce56 executes diverse ω-hydroxylation. FEBS J.

