# Supplementary material for: Three-steps in one-pot: whole-cell biocatalytic synthesis of enantiopure (+)- and (−)-pinoresinol via kinetic resolution
Source: Microb Cell Fact. 2016 May 9;15:78. doi: 10.1186/s12934-016-0472-0 (PMC4862135; doi:10.1186/s12934-016-0472-0)
Supplement: Supplementary file 1 — 10.1186/s12934-016-0472-0 DNA-sequences of the synthetic genes (syatprr2 and syfiplr), plasmids and strains used within this study. [file 12934_2016_472_MOESM1_ESM.pdf]

Additional file 1: DNA-sequences of the synthetic genes (*syatprr*2 and *syfiplr*), plasmids and strains used within this study.

syatprr2 (NCBI Reference Sequence NP_193102.1):

ATGAGTGGAGTCCATTTTCGCACGCACAATATTCCGGTCCAACTTAAGTTAGTCGCAGCC
ATCAAAGAAGCCGGCAACGTGAAACGCTTCCTGCCCAGTGAGTTTGGCATGGATCCTAGC
CGCATGGGCCATGCAATGCCGCCAGGTAGCGAAACGTTCGATCAGAAAATGGAGATCCGT
AATGCCATCAAAGCAGCGGGCATCTCCCATACCTATTTAGTCGGCGCTTGCTTTGCGGCC
TATTTCGGTGGCAATCTGTCTCAGATGGGAACCCTCTTTCCGCCGAAGAACAAAGTGGAC
ATTTATGGCGATGGGAACGTGAAGGTAGTATTTGTCGACGAAGATGACATGGCGAAATAC
ACCGCGAAAACGCTGAATGACCCACGTACCCTGAACAAAACAGTCTATGTACGTCCTACG
GATAACATCTTGACCCAGATGGAACTTGTGCAGATTTGGGAGAAACTGACCGAGAAAGAA
CTGGAAAAGACGTATGTGAGCGGTAATGACTTTCTGGCCGATATCGAAGATAAAGAGATT
TCGCATCAAGCTGGCTTAGGTCACTTCTACCACATTTACTACGAAGGCTGCCTGACCGAT
CACGAAGTTGGTGACGATGAAGAAGCTACTAAACTGTATCCGGATGTGAAATACAAGCGC
ATGGACGAATATCTGAAAATTTTCGTCTGA

syfiplr (GenBank AAC49608.1):

ATGCTGATTAGCTTCAAAATGCAAGGTGCGCATCTGGTGAGTGGCTCCTTTAAAGACTTC
AATAGCCTCGTTGAAGCGGTAAAACTGGTAGATGTGGTTATTTCGGCAATCAGTGGCGTG
CATATCCGCAGCCATCAGATTCTGTTACAGCTGAAACTTGTGGAAGCGATCAAAGAAGCT
GGTAACGTTAAGCGCTTCTTACCGTCTGAATTCGGGATGGATCCTGCTAAATTCATGGAT
ACCGCTATGGAACCCGGGAAAGTCACGTTAGACGAAAAGATGGTGGTACGCAAAGCCATT
GAGAAAGCAGGAATCCCGTTTACCTATGTGTCAGCCAATTGCTTTGCCGGCTATTTTCTG
GGAGGTCTGTGTCAGTTTGGGAAGATCCTCCCATCACGGGATTTCGTCATCATTCATGGC
GATGGGAACAAGAAAGCGATTTACAATAACGAGGATGACATTGCAACTTACGCGATCAAA
ACGATCAATGATCCGCGTACTTTGAACAAAACCATCTACATTAGCCCACCTAAGAACATC
CTTAGCCAACGCGAAGTTGTGCAGACATGGGAGAAACTGATTGGCAAAGAATTGCAGAAA
ATTACGTTGTCGAAGGAGGACTTTCTGGCATCGGTGAAGGAACTGGAGTATGCGCAGCAA
GTGGGTCTGAGTCACTATCATGATGTCAACTACCAGGGATGCTTAACCTCCTTTGAAATT
GGCGATGAAGAGGAAGCCTCTAAACTGTATCCGGAAGTGAAATACACCTCTGTTGAAGAA
TACCTTAAACGCTATGTTGGCGGTCATCACCATCATCACCACTGA

Strains and plasmids used within this study

Strain or plasmid	Characteristics	Source or reference
E. coli DH5α	F$^-$ Φ80*lac*ZΔM15 Δ(*lac*ZYA-*arg*F) U169 *rec*A1 *end*A1 *hsd*R17(r$_k^-$, m$_k^+$) *pho*A *sup*E44 *thi*-1 *gyr*A96 *rel*A1 λ$^-$	Invitrogen
E. coli BL21(DE3*)*	F$^-$ *ompT hsdS*$_B$(r$_B^-$ m$_B^-$) *gal dcm* (DE3)	Novagen
E. coli OverExpress C41(DE3*)*	F$^-$ *ompT hsdS*$_B$(r$_B^-$ m$_B^-$) *gal dcm* (DE3)	Lucigen
E. coli OverExpress C43(DE3*)*	F$^-$ *ompT hsdS*$_B$(r$_B^-$ m$_B^-$) *gal dcm* (DE3)	Lucigen
E. coli SHuffle® T7 Express	*fhuA2 lacZ::T7 gene1* [lon] *ompT ahpC gal λatt*::pNEB3-r1-*cDsbC* (Spec^R, *lacI^q*) Δ*trxB sulA11 R(mcr-73::miniTn10*--Tet^S)2 [dcm] *R(zgb-210::Tn10* --Tet^S) *endA1 Δgor* Δ*(mcrC-mrr)114::IS10*	NEB
pET16b_cgl1	Plasmid containing *cgl*1	E. Ricklefs *et al*., *J Biotechnol* **2014**, *191*, 46–53.
pET22ssl1	Plasmid containing *ssl*1 with a N-terminal His$_6$-tag	M. Gunne, V. B. Urlacher, *PLoS ONE* **2012**, *7*, e52360
pETK316N/D500G	Plasmid containing *cot*A mutant	K. Koschorreck *et al*., *BMC Biotechnol.* **2009**, *9*, 12.
pET28b_psvao	Plasmid containing *psvao* with N-terminal His$_6$-tag	Newlandè von Rooyen, PhD thesis, University of the Free State, Bloemfontein, **2012**
pACYC$_{tac}$_psvao	Plasmid containing *psvao*	This study
pCDF-Duet_syatprr2_his$_6$	Plasmid containing *syatprr2* with C-terminal His$_6$-tag	This study
pCDF-Duet_syfiplr	Plasmid containing *syfiplr*	This study
pCDF-Duet_syfiplr_his$_6$	Plasmid containing *syfiplr* with C-terminal His$_6$-tag	This study
pCDF-Duet_psvao_syatprr2_his$_6$	Plasmid containing *psvao* and *syatprr2*	This study
pCDF-Duet_psvao_syfiplr	Plasmid containing *psvao* and *syfiplr*	This study
