# Supplementary material for: Three-steps in one-pot: whole-cell biocatalytic synthesis of enantiopure (+)- and (−)-pinoresinol via kinetic resolution
Source: Microb Cell Fact. 2016 May 9;15:78. doi: 10.1186/s12934-016-0472-0 (PMC4862135; doi:10.1186/s12934-016-0472-0)
Supplement: Supplementary file 2 — 10.1186/s12934-016-0472-0 Formation of lariciresinol 4 or secoisolariciresinol 5 from pinoresinol 3 in the reactions with cleared cell lysates of different E. coli strains expressing the reductase AtPrR2 (a) or the reductase FiPLR (b). Reaction conditions: 50 mM KPi-buffer, pH 7.5, 0.2 mM pinoresinol 3, 50 µl cleared cell lysate containing AtPrR2 or FiPLR, 0.2 mM NADPH. Optionally, 3 U ml−1 GDH and 20 mM D-glucose were added for cofactor regeneration (cr). The reactions were incubated at 25 °C for 16 h. Besides formation of lariciresinol 4 or secoisolariciresinol 5, no other reaction products were observed. [file 12934_2016_472_MOESM2_ESM.pdf]

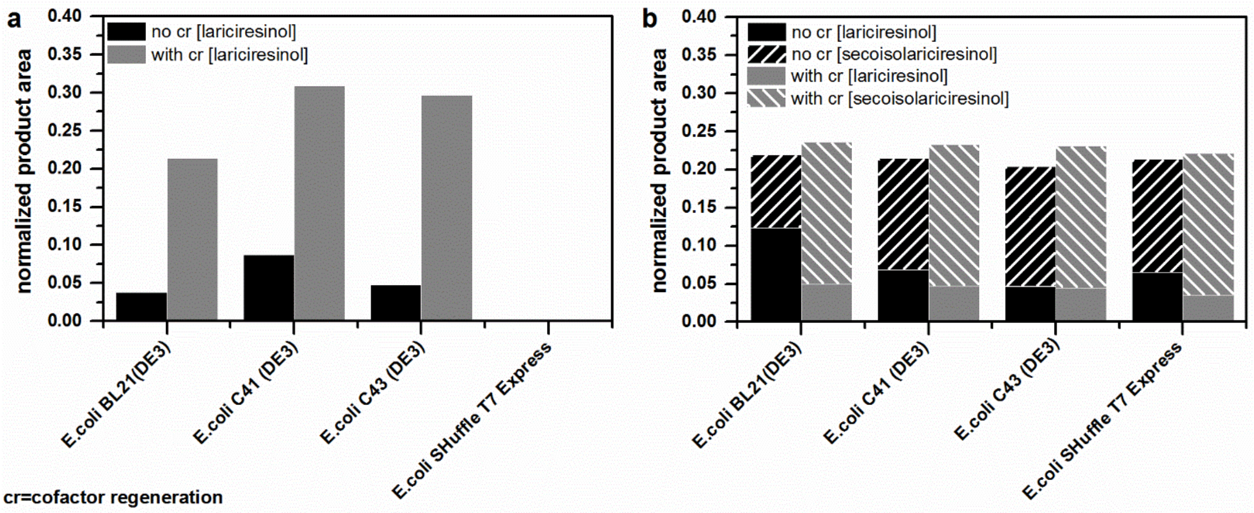

Additional file 2: Formation of lariciresinol **4** or secoisolariciresinol **5** from pinoresinol **3** in the reactions with cleared cell lysates of different *E. coli* strains expressing the reductase AtPrR2 **(a)** or the reductase FiPLR **(b)**. Reaction conditions: 50 mM KPi-buffer, pH 7.5, 0.2 mM pinoresinol **3**, 50 µl cleared cell lysate containing AtPrR2 or FiPLR, 0.2 mM NADPH. Optionally, 3 U ml^{-1} GDH and 20 mM D-glucose were added for cofactor regeneration (cr). The reactions were incubated at 25°C for 16 h. Besides formation of lariciresinol **4** or secoisolariciresinol **5**, no other reaction products were observed.
