# Supplementary material for: Three-steps in one-pot: whole-cell biocatalytic synthesis of enantiopure (+)- and (−)-pinoresinol via kinetic resolution
Source: Microb Cell Fact. 2016 May 9;15:78. doi: 10.1186/s12934-016-0472-0 (PMC4862135; doi:10.1186/s12934-016-0472-0)
Supplement: Supplementary file 3 — 10.1186/s12934-016-0472-0 Cell growth of E. coli C41(DE3) cells co-transformed with pACYCtac_psvao and pET16b_cgl1 in the presence of different concentrations of eugenol 1. Cell cultures were grown at 37 °C, 180 rpm to an OD600 of 0.6 and harvested (indicated by an arrow). Cell pellet was resuspended in TB medium, eugenol 1 was added and the cultures were incubated at 37 °C, 180 rpm. Cell growth was monitored at 600 nm. Black square: 0 mM eugenol 1, blue circle: 1 mM, green triangle: 2.5 mM, purple square: 5 mM, orange circle: 10 mM. [file 12934_2016_472_MOESM3_ESM.pdf]

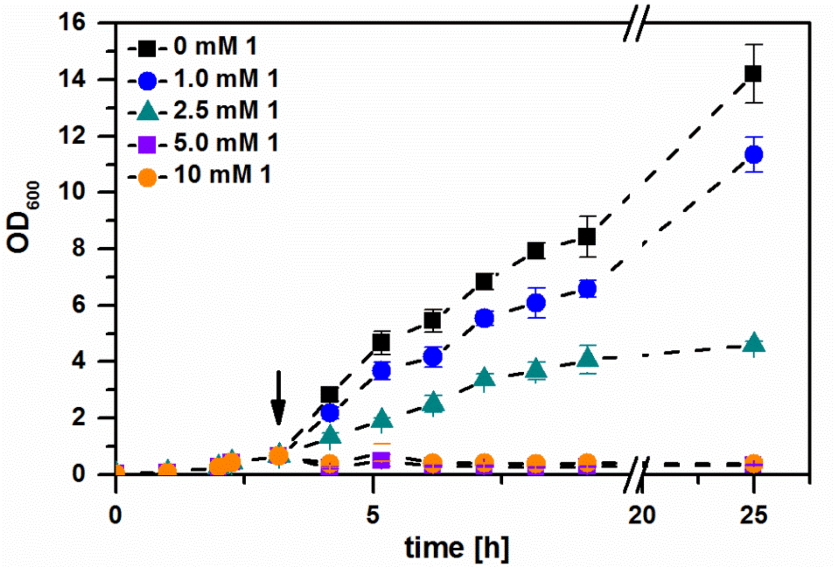

Additional file 3: Cell growth of *E. coli* C41(DE3) cells co-transformed with $pACYC_{tac}$_psvao and pET16b_cgl1 in the presence of different concentrations of eugenol **1**. Cell cultures were grown at 37°C, 180 rpm to an OD_{600} of 0.6 and harvested (indicated by an arrow). The cell pellet was resuspended in TB medium, eugenol **1** was added and the cultures were incubated at 37°C, 180 rpm. Cell growth was monitored at 600 nm. Black square: 0 mM eugenol **1**, blue circle: 1 mM, green triangle: 2.5 mM, purple square: 5 mM, orange circle: 10 mM.
