# Supplementary material for: Three-steps in one-pot: whole-cell biocatalytic synthesis of enantiopure (+)- and (−)-pinoresinol via kinetic resolution
Source: Microb Cell Fact. 2016 May 9;15:78. doi: 10.1186/s12934-016-0472-0 (PMC4862135; doi:10.1186/s12934-016-0472-0)
Supplement: Supplementary file 4 — 10.1186/s12934-016-0472-0 Influence of an energy source on conversion of coniferyl alcohol 2 by E. coli cells. 10 ml E. coli C41(DE3) pCDF-Duet resuspended in reaction buffer (70 g l−1 cww) were supplemented with 1 mM coniferyl alcohol 2. Optionally, 25 g l−1 glycerol or 20 g l−1 D-glucose was added. The reaction was carried out for 24 h at 25 °C, 140 rpm. [2] = coniferyl alcohol 2, [6] = coniferyl aldehyde 6, [IS] = internal standard FSME, * = unspecific peak; chromatograms are shifted vertically. [file 12934_2016_472_MOESM4_ESM.pdf]

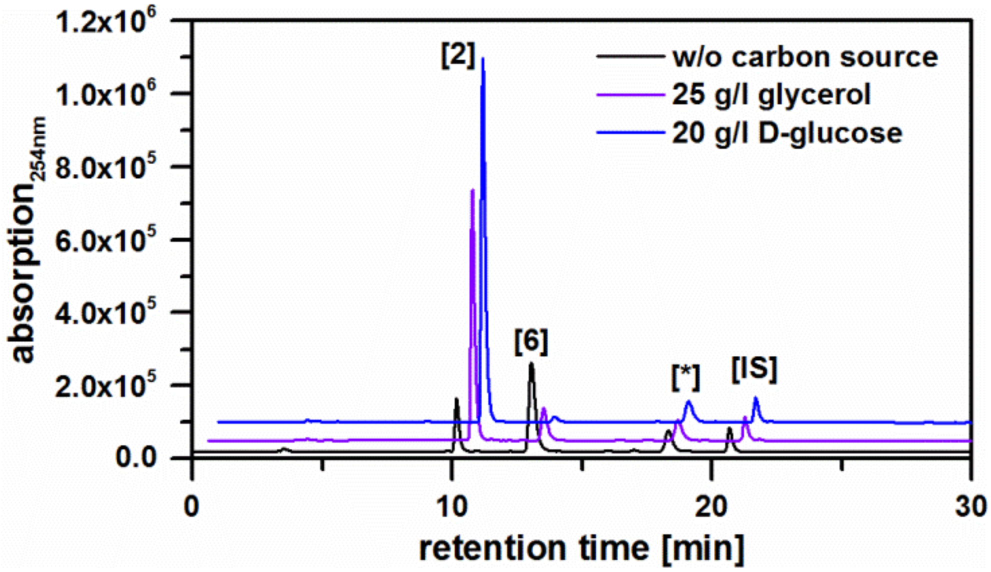

Additional file 4: Influence of an energy source on conversion of coniferyl alcohol **2** by *E. coli* whole-cells. 10 ml *E. coli* C41(DE3) transformed with pCDF-Duet resuspended in reaction buffer (70 g l^{-1} cww) were supplemented with 1 mM coniferyl alcohol **2.** Optionally, 25 g l^{-1} glycerol or 20 g l^{-1} D-glucose was added. The reaction was carried out for 24 h at 25°C, 140 rpm. [2] = coniferyl alcohol **2**, [6] = coniferyl aldehyde **6**, [IS] = internal standard FSME, * = unspecific peak; chromatograms are shifted vertically.
