# Supplementary material for: Three-steps in one-pot: whole-cell biocatalytic synthesis of enantiopure (+)- and (−)-pinoresinol via kinetic resolution
Source: Microb Cell Fact. 2016 May 9;15:78. doi: 10.1186/s12934-016-0472-0 (PMC4862135; doi:10.1186/s12934-016-0472-0)
Supplement: Supplementary file 5 — 10.1186/s12934-016-0472-0 HPLC chromatograms of enantiomeric separations of reaction products. a Application of AtPrR2; b application of FiPLR. [3a] = (+)-pinoresinol 3a, [3b] = (−)-pinoresinol 3b, [4a] = (+)-lariciresinol 4a, [4b] = (−)-lariciresinol 4b, [5a] = (−)-secoisolariciresinol 5a. [file 12934_2016_472_MOESM5_ESM.pdf]

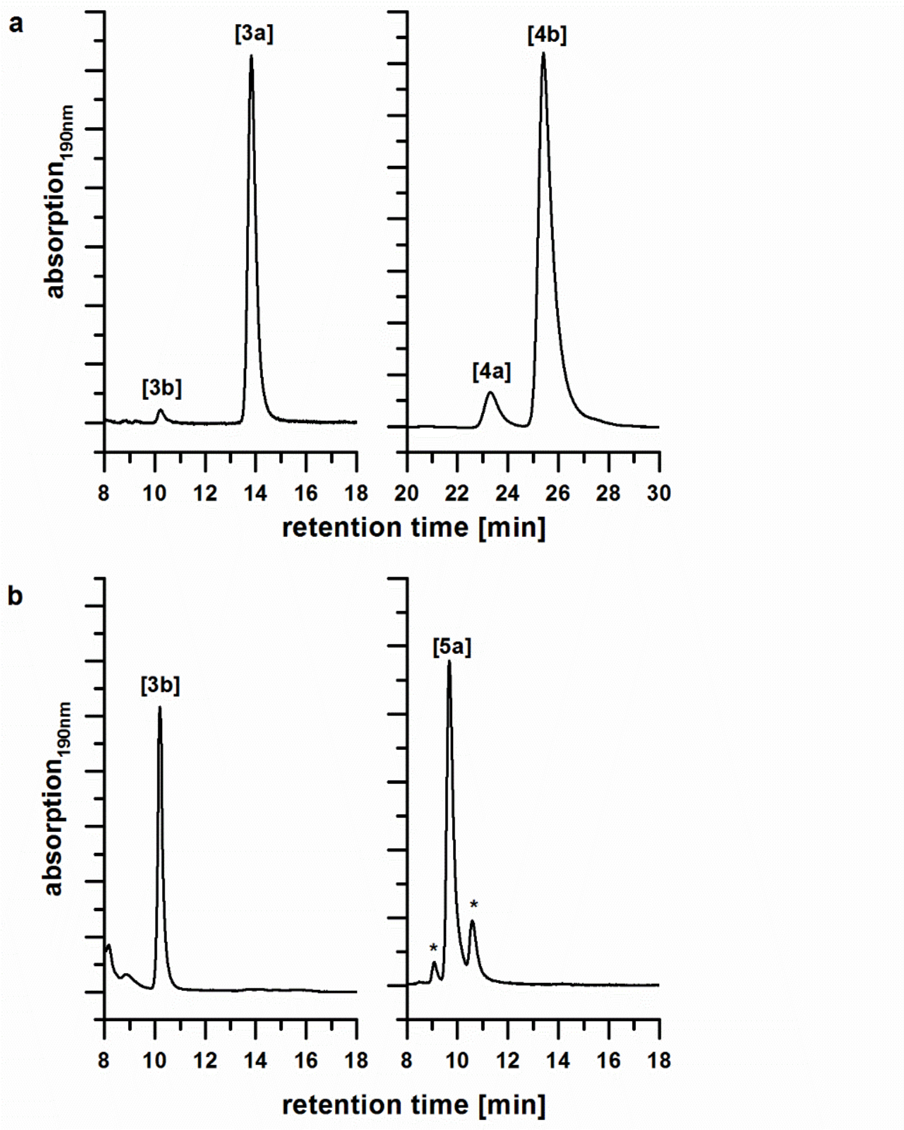


**Additional file 5:** HPLC chromatograms of enantiomeric separations of reaction products. **a** Application of AtPrR2; **b** application of FiPLR. [3a] = (+)-pinoresinol **3a**, [3b] = (-)-pinoresinol **3b**, [4a] = (+)-lariciresinol **4a**, [4b] = (-)-lariciresinol **4b**, [5a] = (-)-secoisolariciresinol **5a**.
