# Supplementary material for: Three-steps in one-pot: whole-cell biocatalytic synthesis of enantiopure (+)- and (−)-pinoresinol via kinetic resolution
Source: Microb Cell Fact. 2016 May 9;15:78. doi: 10.1186/s12934-016-0472-0 (PMC4862135; doi:10.1186/s12934-016-0472-0)
Supplement: Supplementary file 6 — 10.1186/s12934-016-0472-0 Product identification by NMR and HR/MS. [file 12934_2016_472_MOESM6_ESM.pdf]

Additional file 6: Product identification by NMR and HR/MS.

Nuclear magnetic resonance (NMR) spectra were recorded on a Bruker Avance III (600 MHz) on CD_3COCD_3 or Cl_3CD solutions using residual solvent as internal standard. For CD_3COCD_3 the chemical shifts were δ = 2.05 ppm and 2.84 ppm in ^{1}H-NMR spectra, δ = 29.84 ppm, and 206.26 in ^{13}C-NMR spectra, for Cl_3CD δ = 7.26 ppm in ^{1}H-NMR spectra, δ = 77.16 ppm in ^{13}C-NMR spectra. Chemical shifts (δ) are reported in parts per million (ppm) relative to the signal of the residual protic solvents.

Coupling constants *J* [Hz] were directly taken from the spectra and are not averaged. Splitting patterns are designated as s (singlet), d (doublet), dd (doublet of doublets), ddd (doublet of doublets of doublets) and m (multiplet). Peak assignment was based on correlation experiments. High-resolution mass spectrometry (HR/MS) was recorded on maXis in the positive mode with a scan from 50-1500 m/z and the following settings: Source Type ESI, Set Capillary 4000 V, Set End Plate Offset -500 V, Nebulizer 0.3 Bar, Dry Heater 180 °C, Dry gas 4.0 l min^{-1}.

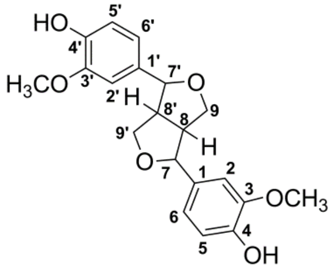

pinoresinol

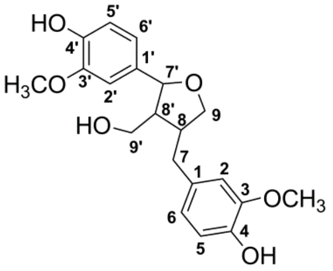

lariciresinol

(+)-Pinoresinol (+)-**3a**: ^{1}H NMR (600 MHz, Acetone-d_6): δ = 6.99 (d, ^3J ($H_{2,2'}$,$H_{6,6'}$) = 1.9 Hz, 2H; 2,2'-H), 6.83 (ddd, ^4J ($H_{5,5'}$,$H_{6,6'}$) = 8.1, ^3J ($H_{2,2'}$,$H_{6,6'}$) = 1.9, ^4J ($H_{6,6'}$,$H_{7,7'}$) = 0.7 Hz, 2H; 6,6'-H), 6.79 (d, ^4J ($H_{5,5'}$,$H_{6,6'}$) = 8.1 Hz, 2H; 5,5'-H), 4.67 (d, ^3J($H_{7,7'}$,$H_{8,8'}$) = 4.4 Hz, 2H; 7,7'-H), 4.22 – 4.18 (m, 2H; 9,9'-H), 3.84 (s, 6H; 3,3'-OCH_3), 3.80 (dd, ^2J = 9.1, ^3J ($H_{8,8'}$,$H_{9,9'}$) = 3.8 Hz, 2H; 9,9'-H), 3.11 – 3.06 (m, 2H); ^{13}C NMR (151 MHz, Acetone-d_6): δ = 148.27 (C-3,3'), 146.77 (C-4,4'), 134.16 (C-1,1'), 119.61 (C-6,6'), 115.46 (C-5,5'), 110.61 (C-2,2'), 86.63 (C-7,7'), 72.21 (C-9,9'),

56.24 (3,3’-OCH_3), 55.24 (C-8,8’); HRMS (ESI, pos.) calculated for $C_{20}H_{22}NaO_6$ $[M+Na]^+$ m/z: 381.1309, found: 381.1304; ee-value: 96%

(-)-Lariciresinol (-)-**4b**: ^{1}H NMR (600 MHz, Chloroform-*d*): δ = 6.90 – 6.66 (m, 6H; ArH), 4.78 (d, *J* = 6.6 Hz, 1H; 7’-H), 4.05 (dd, *J* = 8.6, 6.6 Hz, 1H; 9-H), 3.90 (dd, *J* = 10.8, 7.1 Hz, 1H; 9’-H), 3.87 (s, 3H; 3’-OCH_3), 3.86 (s, 3H; 3-OCH_3), 3.78 – 3.69 (m, 2H; 9’-H, 9-H), 2.91 (dd, *J* = 13.6, 5.2 Hz, 1H; 7-H), 2.77 – 2.69 (m, 1H, 8-H), 2.54 (dd, *J* = 13.6, 10.7 Hz, 1H; 7-H), 2.43 – 2.37 (m, 1H; 8’-H); ^{13}C NMR (151 MHz, Chloroform-*d*): δ = 146.77 (C-3’), 146.66 (C-3), 145.15 (C-4’), 144.11 (C-4’), 134.89 (C-1’), 132.40 (C-1), 121.31 (C-6), 118.87 (C-6’), 114.56 (C-5), 114.33 (C-5’), 111.35 (C-2), 108.46 (C-2’), 82.94 (C-7’), 73.01 (C-9), 60.99 (C9’), 56.05 (2xOCH_3), 52.72 (C-8’), 42.53 (C-8), 33.42(C-7); HRMS (ESI, pos.) calculated for $C_{20}H_{24}NaO_6$ $[M+Na]^+$ m/z: 383.1465, found: 383.1464; ee: 85%

HR/MS results: HR/MS acquisition parameters: (ESI, pos.) m/z calculated for $[M+Na]^+$.

Product No.	Measured m/z	Ion Formula	Calculated m/z	err [ppm]
**3**	381.1304	$C_{20}H_{22}NaO_6$	381.1309	1.2
**4**	383.1464	$C_{20}H_{24}NaO_6$	383.1465	0.4
**5**	385.1624	$C_{20}H_{26}NaO_6$	385.1622	-0.5
